# Lack of Rigour in the Review. Comment on Kuligowski et al. Manual Therapy in Cervical and Lumbar Radiculopathy: A Systematic Review of the Literature. *Int. J. Environ. Res. Public Health* 2021, *18*, 6176

**DOI:** 10.3390/ijerph192315613

**Published:** 2022-11-24

**Authors:** Pawel Posadzki

**Affiliations:** Kleijnen Systematic Reviews Ltd., 6 Escrick Business Park, Escrick, York YO19 6FD, UK; pawel.posadzki@hotmail.com; Tel.: +447-470-737-169

I read with interest the article by Kuligowski et al., 2021 published in the Journal [1]. However, there are number of methodological weaknesses embedded in the review, and its contribution to the evidence base is questionable.

First and foremost, I will start with Prof Altman’s classic: “we need less research, better research, and research done for the right reasons” [2]. This quote is more relevant than ever and speaks to the very poor rationale of the review by Kuligowski et al., 2021. The authors failed to acknowledge the existing Cochrane and non-Cochrane systematic reviews in the field [3,4,5]. Hence the authors’ claims that “(reviews) regarding the lumbar region are minimal” or “(…) a limited number of RCTs (randomized controlled trials) was found to be eligible” in this review is far from the truth.

- Without going into too many details, similar systematic reviews [3,4] (in terms of populations, interventions, comparators, outcome measures and study designs (PICO) but only focusing on low back pain) included 51 and 47 studies on the topic, respectively, compared with only six by Kuligowski et al., 2021—even though these were published in 2018 and 2019, respectively (presumably a dozen more RCTs were published since then and 2021). There are an additional 20 studies evaluating Mulligan mobilizations with movement in the treatment of low back pain, [6] dozens more on neuromobilizations [7], 38 on massage (as of 2015), etc. [5]. Given the breadth of the eligibility criteria and PICO, lack of date restrictions, we should be looking at a review with >200 studies (not 27 as Kuligowski et al., 2021 are claiming).
- The searches were not comprehensive and did not include an appropriate range of databases, e.g., Embase, Cochrane Central, PsycINFO, CINAHL, SCOPUS and AMED were omitted.
- Using the search terms provided by the authors, there were 50,343 ‘hits’ in PubMed and 59,111 randomized trials in the Cochrane Library itself (date queried: 17/10/2022)—therefore only 473 records, i.e., 0.93%, sounds highly improbable and certainly not impressive.

Appropriately designed search strategy is a foundation of every systematic review. Unsurprisingly then, not including all the relevant terms such as radiculopathy, low back pain, pain associated with spondylosis, sacroiliac joint syndrome, trauma-induced, disc herniation, or pelvic anteversion resulted in poor yield/retrieval (in terms of population). In terms of intervention one would encourage the authors to use other relevant terms (and their derivates) such as high velocity thrust, low amplitude thrust (HVLA), low velocity thrust, low amplitude thrust (LVLA), massage, Trigger Point Therapy, Positional/myofascial Release Techniques, Rolfing, etc. In addition, considering other than conceptual and practical frameworks such as Cyriax, McKenzie, Lewit or Shacklock would strengthen the methods, results and conclusions. Not including all these search terms and keywords suggests that Kuligowski et al., 2021 are not experts in manual therapy (MT) but merely observers of the field. On related note, MT is poorly, if at all defined by the authors. MT is physical treatment primarily used by physical therapists, physiotherapists, occupational therapists (but also chiropractors, osteopaths, alternative medicine practitioners, massage therapists, etc.) to treat musculoskeletal pain and disability [8]. The authors also included complex interventions, e.g., manual traction plus exercise and massage, hence attributing the effects of interventions (if any) solely to MT is unjustified.

There are several logical/conceptual fallacies which render internal validity very uncertain, and the eligibility criteria were vague and rather inappropriate to address the review’s question. For example, presence of radiculopathy (for lumbosacral region) was their inclusion criterion. However, as per Table 1 (among other places), they included 21 trials of cervical radiculopathy.

There are no comparators listed under eligibility criteria, hence the reader simply does not know whether all types of control groups were admissible; and which arms were used as control groups in cases of three arm trials. More importantly, the authors did not specify primary or secondary outcomes for their review (typically pain, disability, function, quality of life, adverse-effects). Hence we do not know what “treating cervical and lumbar radiculopathy” actually means.

Rather insufficient study characteristics were considered to be able to interpret the results and were extracted for use in the synthesis. For instance, no numbers are reported in Table 1 (or in the text) such as means, standard deviations, *p*-values, confidence intervals, etc. Vis-a-vis the absent numbers, the (narrative) synthesis part of the review is missing almost entirely; the authors only list the studies and present results of the Pedro scale (methodological concerns). It is also unclear why the authors used the Pedro score (almost 20 years old) when newer and much more commonly used tools are available, i.e., Cochrane ROB or ROB-2 [9].

Currently, the robustness of the findings is poor. In addition to performing subgroup and/or sensitivity analyses, the authors should also add the Grading of Recommendations, Assessment, Development and Evaluation (GRADE) approach to evaluate the certainty of the evidence, i.e., confidence in the effect estimates.

Finally, there several statements which are difficult to fully comprehend e.g., “Due to the controversial homogeneity of the manual therapy methods used and the specific aim of this paper, we decided not to design our study as a meta-analysis”. Presumably the authors meant clinical, methodological or/and statistical heterogeneity (not homogeneity); and undertaking a meta-analysis in any systematic review depends on similarity of PICOS. Others, i.e., “A small number of LR clinical trials was also a significant barrier in unifying treatment methods for this pathology” are rather incomprehensible, despite the best efforts of this reader. I realize that English is not the authors’ first language, however, this could have been addressed at the peer-review/proofreading stage(s). The authors add even more confusions by adding the following (poorly phrased) statements to the conclusions: “Exercise programs itself are efficient and improve patients’ outcomes, but there is no standardization of specific activities to specific pathology algorithm”- which is not aligned with the study objectives, and eligibility criteria.

In summary, there are some serious concerns with the data collected; the study is virtually impossible to replicate. The cornerstone of evidence synthesis is to include and critically evaluate the totality of the evidence. However, when tens of relevant studies have been missed the credibility of the findings is very uncertain. This should be a systemic review which adds very little to the evidence base (if anything) and increases the research waste. Most probably, undertaking an overview of the existing systematic reviews (which the authors missed when formulating their rationale) in the field would be much more informative for the readers, clinicians, patients and policymakers alike.
